# COVID-19 and cancer: insights into their association and influence on genetic and epigenetic landscape

**DOI:** 10.2217/epi-2023-0052

**Published:** 2023-05-02

**Authors:** Mrinal K Ghosh, Sunny Kumar, Kirat K Ganguly, Pratyay Ghosh, Shaheda Tabassum, Bhaskar Basu, Malini Basu

**Affiliations:** 1 Cancer Biology & Inflammatory Disorder Division, Council of Scientific & Industrial Research-Indian Institute of Chemical Biology (CSIR-IICB), TRUE Campus, CN-6, Sector V, Salt Lake, Kolkata, 700091 & 4, Raja S.C. Mullick Road, Jadavpur, Kolkata, 700032, India; 2 Department of Microbiology, Michael Madhusudan Memorial College, Durgapur, 713216, India; 3 Department of Electronics & Instrumentation Engineering, Techno India College (Main), EM-4/1, Sector-V, Salt Lake, Kolkata, 700091, India; 4 Department of Microbiology, Dhruba Chand Halder College, University of Calcutta, Kolkata, 743372, India

**Keywords:** cancer, COVID-19, epigenetics, genetics, immune response, SARS-CoV-2 pandemic

## Abstract

The COVID-19 outbreak has created disaster globally, and mankind is yet to come in terms with combating this global menace. Amid this turmoil, immunocompromised individuals like cancer patients exhibit dismal immune responses toward such infection. In order to treat cancer patients during such adverse situations, it is necessary to understand the phenomena that interlink these two diseased states. Modulation of host epigenetic landscape is a key hallmark of both cancer and viral infections, including COVID-19. Our review aims to shed light upon the interplay between COVID-19 and cancer, primarily through the genetic and epigenetic modulations of the gene expression profile, so as to design better therapeutic strategies in the near future.

COVID-19 is caused by the positive-strand, 29-kb ssRNA virus SARS-CoV-2. Many recent reports have well established the mechanism of entry of this virus to the host via the ACE2 receptor, which mostly occurs in type II lung epithelium. The virus uses the spike protein RBD, containing a basic main structure and a receptor-binding domain, to associate with the extrinsic layer of the ACE2 receptor for entry [1]. Molecular affinity between ACE2 and the spike protein is a significant factor for tissue tropism associated with disease etiopathogenesis. The SARS-CoV-2 spike protein is broken down into two fragments (S1 and S2) by proteases (CTSL, TMPRSS2 and furin). Upon host entry via endocytosis, the genomic RNA is uncoated and translated into effector proteins responsible for subsequent replication and assembly of new virus particles. Newly assembled viral particles are finally secreted out of the infected cells by exocytosis and thereafter they infect neighboring host cells. The host system responds by significantly escalating the secretion of several chemokines and cytokines, termed as the cytokine storm, which in turn causes increased macrophage infiltration at the site of infection and initiation of pyroptosis [2]. Alveolar flexibility of the lungs is reduced by the accumulation of broncheoalveolar fluid, which in turn produces symptoms such as breathing difficulties and pneumonia, and in serious cases can lead to multiorgan dysfunction [3–5]. This infection is linked with disproportionate levels of anti-thrombin III, fibrinogen and D-dimers due to the cytokine storm, which may lead to increased coagulopathy [6]. As a result, venous thromboembolism pulmonary congestion and lung microvascular thrombosis are quite common in COVID-19 patients [7].

In general, most of the coronaviruses are impotent in accessing the host genome, but they might be able to inhibit the host epigenome through altered cellular signaling dynamics. Recent research has been concentrated on how viral pathogens employ the molecular basis of epigenetic mechanisms to permit their spread, establishment and determination. It is now possible to outline the epigenetic landscape and assess the disease progression at a genome-wide scale using advanced high-throughput sequencing technology. However, the access to advanced technologies and improved resolution – including epigenetics, 3D genome structure profiling and changes in genetic expression in both virus and host cells at the single-cell level – has immediately transfigured this area and found a specific chromatin map that suggests a prevalent relationship between epigenetic pathways and genetic expression in communicable diseases including COVID-19 [8].

Among various diseases, cancer is a major serious health problem around the globe. Amid the growing wildfire of coronavirus engulfing mankind, it is a matter of great concern to manage the therapeutic regimen for immunocompromised cancer patients if they get infected with this deadly virus. The intensity of this affliction is greater in cancer patients, with higher lethality rates. Thus it is imperative to establish the molecular inter-relationships between cancer and COVID-19 in order to develop proper treatment regimens for patients afflicted with both these pathological conditions [9]. In this report, we have attempted to provide a comprehensive study into this double-edged dilemma of current times.

## Impact of SARS-CoV-2 on cancer patients’ prognosis

It has long been established that there exists a key link between viral infections and cancer development. This suggests that metabolic regulation of transformed cells may affect the patient’s prognosis in relation to SARS-CoV-2 [10]. According to one study, nearly 15% of all human cancers have been attributed to host infection by viruses [11]. In another report by the National Cancer Institute, pathogens (including both DNA- and RNA-containing viruses) have been implicated in one out of every four cancer cases in developing nations and in one out of ten cases in developed nations [12]. Viruses also pose the threat of increasing complications in cancer-afflicted patients, thereby significantly impairing patient prognosis. Viruses responsible for respiratory tract infections most likely pose the greatest risk of advancing complications in cancer patients. As an example, during the Middle East respiratory syndrome (MERS) outbreaks in 2012 and 2015, cancer patients showed a significantly greater rate of mortality following infection with MERS-CoV [13]. Similarly, SARS-CoV-2 infections have been found to be far more effective in severely affected cancer patients compared to non-cancer patients and have greatly increased the likelihood of these patients requiring invasive ventilation [14]. One of the earlier reports on the topic carried out in Wuhan stated that out of a series of 28 SARS-CoV-2-infected cancer patients, 15 had developed serious complications, while eight did not survive. Notably, out of the 28 patients, ten displayed signs of metastatic disease. The causes of death were stated to be acute respiratory distress syndrome (ARDS), septic shock and myocardial infarction. The link between SARS-CoV-2 infection and increased severity in cancer patients can be better understood by following the viral life cycle and understanding the impact of infection on the patient’s health at the molecular level [15].

Interestingly enough, many of the molecular events associated with SARS-CoV-2 infection, such as the cytokine storm and the increased incidence of coagulopathy, are also observed in cancer patients. Inflammation is a major factor that induces and drives cancer progression and metastasis. It enhances lung cancer epithelial cells’ proliferation and migration via STAT3 signaling [15]. It has been found that around 15–20% of all cancers are preceded by infection, chronic inflammation or autoimmune disorders at the same organ site [16–18]. The area of the body consisting of the tumor, the surrounding stromal tissue and accumulated inflammatory cells is termed the tumor microenvironment. This site is proinflammatory in nature, with inflammatory molecules serving as direct growth-stimulating and morphogenic factors. Tumor cells located within this site are in a continuous state of plasticity, which is essential for tumor progression. Hence the proinflammatory cytokine storm initiated during severe SARS-CoV-2 infection can serve to aggravate conditions in cancer patients by further strengthening the proinflammatory conditions within the tumor microenvironment [19]. However, direct evidence supporting this idea is lacking and requires further investigation. ACE2 expression is upregulated in some cancers such as renal, pancreatic and lung carcinomas, and stage-dependent variations in ACE2 expression are also observed. As a result, patients with ACE2-overexpressing cancers are highly susceptible to this infection, and the severity of such infection is expected to be much greater as compared with non-cancer patients. Similarly, TMPRSS2 expression is highly upregulated in prostate cancer, but only moderately upregulated in lung and renal cancers; thus the risk of prostate cancer patients contracting COVID-19 is greater than that for lung cancer patients [14,20,21]. This will be explained in further detail in the following section. Coagulopathy is yet another similarity between COVID-19 and cancer. Coagulation events such as disseminated intravascular coagulation are common in cancer patients; this is mostly attributed to the release of cancer-associated factors (e.g., plasminogen activator factor, proinflammatory cytokines, tissue factors and mucins) that greatly enhance the threat of thrombosis [22]. Certain cancers (e.g., ovarian and lung cancers) exhibit greater risk of coagulation as compared with other cancers such as breast cancer. Hence the contraction of COVID-19 by cancer patients who are already susceptible to experiencing coagulation events greatly impairs their prognosis [20,23].

Based upon recent literature, focused, in-depth studies are still required to understand the impact of COVID-19 on cancer patients. Treatment of SARS-CoV-2-infected cancer patients is quite difficult, and a lot of unsolved mysteries remain about COVID-19 affected cancer patients who are receiving chemotherapy/radiotherapy; for example, is it safe for cancer patients with COVID-19 to receive chemo/radiotherapy along with anti-COVID therapy? Furthermore, the mortality and morbidity rates of patients with both diseases are still unsolved or not clear yet. Thus more clinical investigations/trials are required to estimate the effect of this infection on the outcomes for cancer patients.

## Inflammatory signaling & immune evasion in COVID-19 are linked with cancer

COVID-19 has serious impacts on cancer patients. Studying these impacts and their inter-relationships is crucial for the treatment and management of cancer patients. In this section we discuss the connections between COVID-19, inflammation and cancer which leads to immune evasion.

### Inflammatory immune responses

Following initial infection of lung alveolar epithelial cells, the immune system gets triggered through the inflammatory route. After entry, the virus is engulfed by antigen-presentation cells (APC), macrophages and dendritic cells (DCs), and presents SARS-CoV-2 processed antigen to T cells, causing their activation and differentiation and the secretion of cytokines. Viral infection is immediately sensed by the host defense system using pattern recognition receptors and Toll-like receptors (TLRs) for recognizing virus-associated molecular patterns (viral proteins, lipids and nucleic acids). Thereafter, different pathways like NF-κB, IRF3 and MAPK are activated, resulting in induction of expression of IL-1β, interferons and IL-6 proinflammatory cytokines. The large-scale uncontrolled cytokine storm generally presents itself in the form of systemic inflammation. IL-6 and IL-1β are the predominant cytokines produced during the early stage of this infection; IL-1β in particular is responsible for promoting inflammation in the alveoli and bronchi of patients exhibiting pulmonary injury. On the other hand, higher levels of IL-6 can stimulate the complement system, which greatly enhances the vascular permeability, thereby giving the circumstances for further spread of the infection. During this, anti-inflammatory factors (type I interferons and IL-10) are generally found to be depleted during such incidences of this infection [24–27].

Clinically, patients with severe SARS-CoV-2 infections have an induced cytokine profile like that induced by SARS-CoV-1 and MERS-CoV [28]. C-reactive protein and erythrocyte sedimentation rate have also been found to be elevated in severe patients; these findings were related to hypercoagulation, ARDS and disseminated intravascular coagulation, and presented as thrombosis, thrombocytopenia and gangrene on the limbs [29,30].

### Interleukin-6

**Figure 1.**
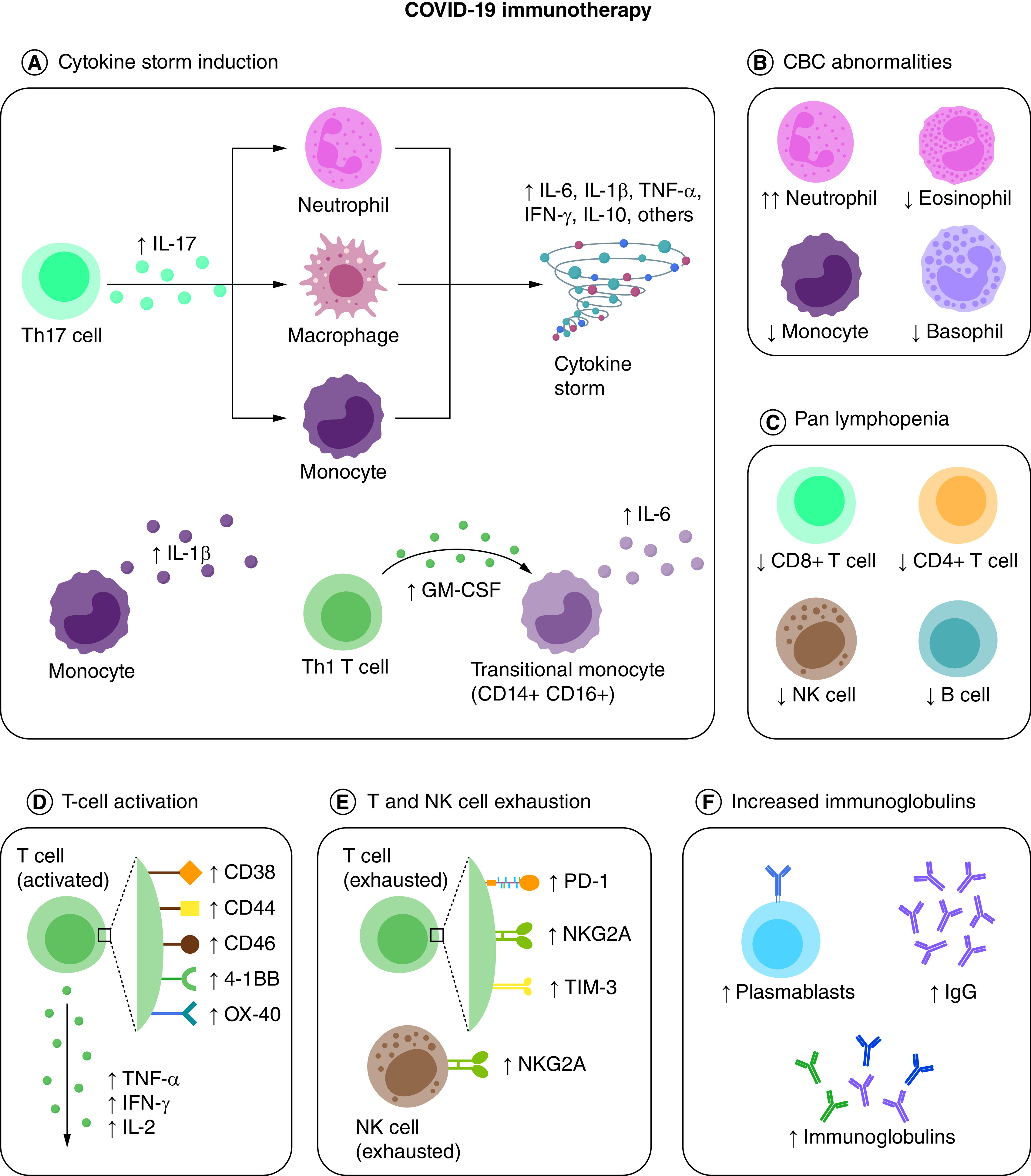
Clinical immunopathology in COVID-19. The figure depicts the various immunological responses and changes observed **(A–F)** in SARS-CoV-2-infected patients.

Available reports also show crosstalk between cancer and SARS-CoV-2 infection from an immunological perspective. IL-6 is reported as one of the major hallmarks of the cytokine storm induced during this infection and is even considered as a key mediator during the transitioning of lung carcinogenesis [31,32]. Increased NF-κB signaling induced by *KRAS* mutation triggers the expression of IL-6 [33]. Overexpressed IL-6 has also been linked with severe SARS-CoV-2 and ARDS. Thus the overexpression of such cancer-associated genes can direct the worse prognosis reported in SARS-CoV-2-infected cancer patients compared with noncancerous patients [34]. To develop better therapeutics and clinical interventions against coexisting COVID-19 and cancer, this pool of data essentially needs to be validated with *in vivo* and further molecular studies (Figure 1).

### Modulation of NF-κB & interferon signaling: paving the way to immune evasion

**Figure 2.**
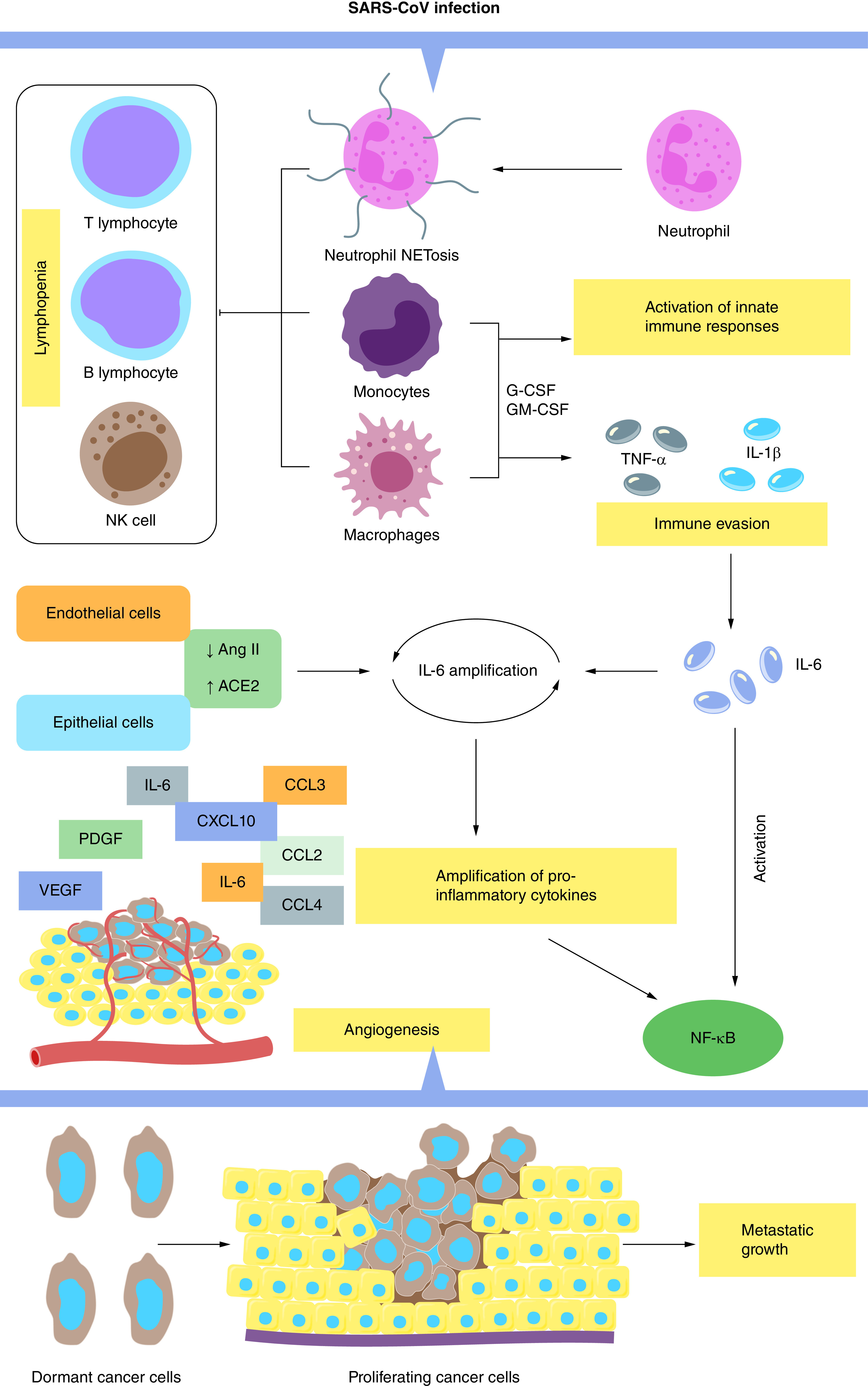
Cancer and COVID-19 interconnection. The figure depicts the role and impact of SARS-CoV-2 upon various immune signatures and their connection in cancer progression.

Cathepsin L (CTSL), trypsin and factor Xa break the spike protein and allows the virus entry [35]. IL-8 release is induced in response to the spike protein in fibroblast and epithelial cells which display ACE2 receptors via the AP-1 pathway [36]. Subsequent evidence supports the essential responsibilities of major regulatory proteins encoded by MERS-CoV that will block NF-κB signaling in order to evade the host defense. Destruction of I-κB has been shown to stimulate the nuclear transportation of NF-κB, which further stimulates the cytokine expression. Activation of Toll-like receptors, retinoic acid-inducible gene-like receptors and nucleic acid sensors identify the antigen-associated molecular recognition impression upon viral attack. MDA5 and RIG1 function was studied through mitochondrial antiviral-signaling protein (MAVS) and was shown to prime the polyubiquitination of I-κB with recruitment of TAK1 and TRAF, in turn inducing NF-κB [37,38]. Moreover, the early evidence proves the emergence in the pathogen of multiple proteases and proteins causing the desensitization of these adaptor molecules, which suppresses NF-κB and elicits immune evasion [39]. A recent study using coronavirus-infected 229E cells revealed that chromatin recruitment of the p65 (RelA) subunit of NF-κB is crucial for proper induction of its target genes. p65 (RelA) occupies enhancer elements and the transcription start site of its own promoter; as identified by enhanced acetylation of H3 and H4 histones, infection leads to increased expression of p65 and its target genes which are associated with antiviral immune responses [40]. Activated NF-κB permits the formation of the A20 biomolecule, needed for effective SARS-CoV-2 viral replication [41,42]. Typically, the recruitment of several inducible transcription factors to enhancer regions having H3K4me1 and H3K27ac promotes the acetylation of H4K5 and H3K36 in chromatin assembly around the promoters to determine the virus-mediated host response. The feasible association of these proteins having distinct DNA methyltransferases (DNMTs) were nicely studied [40]. Menachery *et al.* found that a feasible accumulation of global H3K27me3 with suppression of interferon-stimulated gene (ISG) expression through DNA methylation was responsible for antigen presentation after H5N1VN1203 and MERS-CoV infections [43]. Upon infection, the feasible association of H3K4me3 and TNF-α is also observed to induce the innate immune responses in monocytes, and antigen presentation of DC1 and T_H_1/T_H_17-mediated immune response [44,45]. However, the enhancement of TNF-α/IFN-γ catalyzes MLL1 and further activates H3K4-mediated methylation, which is vital for DC polarity [46]. Recently, a key role of the NS1 influenza virus was studied in relation to the regulation of the JAK–STAT signaling pathway, with the promotion of DNMT3b transport from the nucleus to the cytosol which further leads to proteasomal degradation by K48-mediated ubiquitination. The effects of certain JAK–STAT signaling suppressors (SOCS1, SOCS3 and PIAS1) are induced by promoter demethylation which leads to the inhibition of interferon signaling (Figure 2) [47–49].

The exploitation of virus-mediated host machinery to encode the viral proteins responsible for impeding the innate immunological responses is essential to demonstrate a successful infection state. In transcriptome methylation, the participation of several RNAs (tRNAs, mRNAs and rRNAs) and other noncoding RNAs is mandatory for modulating the gene expression profiles. Systematically, the capping of 5′-guanosine in eukaryotic mRNA occurs at 2′-O positions by methyltransferases to differentiate the intrinsic self-capped RNA from extrinsic non-self RNA encoded by viruses lacking methylated nucleotides. These mechanistic details have been well established, and it is suggested that IFIT1 regulates this effect by specifically associating with viral RNAs that are lacking in 2′-O-methylation at their 5′ end, resulting in inhibition of viral RNA translation. A seminal investigation suggests that multiple types of viral pathogens (SARS-CoV-2, Japanese dengue, encephalitis, flaviviruses, mouse, coronavirus and vaccinia) adopt this mechanism for the establishment of infection and immune evasion. Additionally, the interacting complex including estrogen, SMYD2 and Hsp90 serves a crucial role in autophagy [50–56]. An identical type of systematic approach may operate in SARS-CoV-2-infected hosts.

The above knowledge about immunological linkage between cancer and COVID-19 helps in the repurposing of drugs. However, the link between COVID-19 and cancer is not solid. As with knowledge of other infections, links between the diseases were also predicted, but how this infection affects the tumor microenvironment at the molecular level is still not known. Thus immunological investigations are still required to solidify the connections between them. Moreover, in-depth molecular-level understanding of immune biomarkers may be implicated or translated into clinical perspectives to design inhibitors against cancer for patients affected with COVID-19.

## ACE2, CTSL/B & TMPRSS2: dual perspectives in COVID-19 & cancer

**Figure 3.**
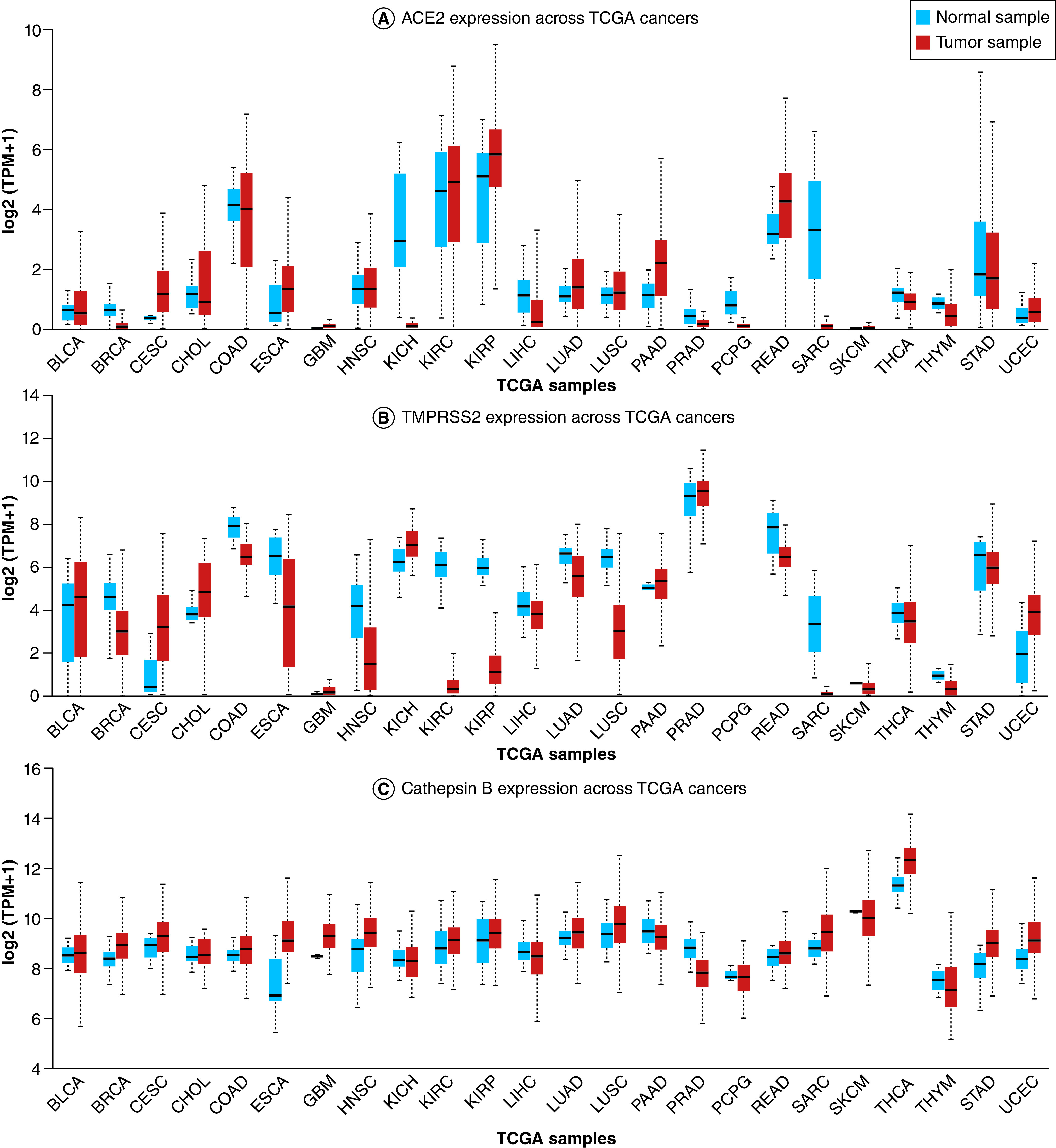
Expression of *ACE2*, *TMPRSS2* and cathepsin B in multiple cancer tissues in The Cancer Genome Atlas. **(A)** Bioinformatics-based pan-cancer analysis representing *ACE2*, **(B)** *TMPRSS2* and **(C)** cathepsin B expression in different cancer tissues. The Cancer Genome Atlas dataset was analyzed using the UALCAN (http://ualcan.path.uab.edu/analysis.html) platform. BLCA: Bladder urothelial carcinoma; BRCA: Breast carcinoma; CESC: Cervical squamous cell carcinoma and endocervical adenocarcinoma; CHOL: Cholangiocarcinoma; COAD: Colon adenocarcinoma; ESCA: Esophageal carcinoma; GBM: Glioblastoma multiforme; HNSC: Head and neck squamous cell carcinoma; KICH: Kidney chromophobe; KIRC: Kidney renal clear-cell carcinoma; KIRP: Kidney renal papillary-cell carcinoma; LIHC: Liver hepatocellular carcinoma; LUAD: Lung adenocarcinoma; LUSC: Lung squamous cell carcinoma; PAAD: Pancreatic adenocarcinoma; PCPG: Pheochromocytoma and paraganglioma; PRAD: Prostate adenocarcinoma; READ: Rectal adenocarcinoma; SARC: Sarcoma; SKCM: Skin cutaneous melanoma; STAD: Stomach adenocarcinoma; THCA: Thyroid carcinoma; THYM: Thymoma; UCEC: Uterine corpus endometrial carcinoma.

The repeated occurrence and poor prognosis of cancer in COVID-19 patients and *vice versa* indicates some putative crosstalk between these two pathological conditions. The transformed immunological landscape, coupled with signaling cascades, plays a pivotal role in the activation and amplification of viral particles and may contribute to the greater vulnerability reported in COVID-19-affected cancer patients. Due to their roles in both coronavirus pathogenesis (SARS, MERS, SARS-CoV-2) and malignancy, host genes such as *ACE2* and *TMPRSS2* are consistently gaining interest. We believe that it is essential to have a proper understanding of the epigenetic alteration status of key genes like *ACE2* and *TMPRSS2* which are frequently reported as the key molecules involved in COVID-19 infection (Figure 3) [57–59].

### ACE2 & CTSL/B

The cleavage of the spike protein is accomplished by host proteases like CTSL/B, resulting in viral entry [60]. Amplified *CTSL* expression was reported among nine different types of tumors: head and neck squamous cell carcinoma, esophageal carcinoma, glioblastoma multiforme, pancreatic adenocarcinoma, lymphoid neoplasm diffuse large B-cell lymphoma, lower grade glioma, thymoma, skin cutaneous melanoma and stomach adenocarcinoma at the transcriptional level by means of the Gene Expression Profiling Interactive Analysis (GEPIA) tool [61–63]. On the other hand, *ACE2* was reported to be overexpressed in pancreatic adenocarcinoma, stomach adenocarcinoma, colon adenocarcinoma, kidney renal papillary cell carcinoma and rectal adenocarcinoma. In stomach adenocarcinoma, CTSL/B protease is overexpressed due to genomic amplification. Co-overexpression of both CTSL/B and ACE2 indicates a greater threat of this infection in these cancers. Therefore, further confirmation and validation is required to solidify the causal modes of ACE2 and CTSL/B co-overexpression, for the better understanding of the relationship between risk of this infection and these groups of cancers [64].

### TMPRSS2

TMPRSS2 is a necessary molecule required for activation of the S protein [65]. Moreover, c-BioPortal analysis found specific mutations in upregulated genes [66]. *TMPRSS2* was established to be highly expressed in prostate adenocarcinoma, cervical squamous cell carcinoma, endocervical adenocarcinoma, kidney chromophobe, colon adenocarcinoma, rectal adenocarcinoma, uterine carcinosarcoma and uterine corpus endometrial carcinoma. The detection of greater expression of *TMPRSS2* in prostate adenocarcinoma was authenticated by its profuse level in secretory prostate epithelium, and *TMPRSS2*–*ERG* genetic fusion is considered to be a signature complex for prostate cancer [67–69].

It should be mentioned that an increased mortality rate was reported in males throughout the COVID-19 pandemic. Similar observations led researchers to hypothesize whether sexual dimorphism is linked to severity of SARS-CoV-2 in patients due to the augmented expression of *TMPRSS2* in males. This dimorphism in combination with amplified androgen receptor expression may make prostate cancer patients greatly susceptible to this infection [69,70]. This hypothesis was further corroborated through the observation that prostate cancer patients receiving androgen-deprivation therapy showed a reduction in infection compared with patients who were not treated or were suffering from another type of cancer [71,72]. Thus, TMPRSS2 should be a logical therapeutic target for intervention among prostate cancer patients infected with SARS-CoV-2 [73]. *TMPRSS2* and *ACE2* expression profiling have been studied in several cancer types (lung adenocarcinoma, colon adenocarcinoma, breast invasive carcinoma, stomach adenocarcinoma, liver hepatocellular carcinoma and prostate adenocarcinoma), where levels were observed and matched with respective non-cancer counterparts using the GEPIA2 platform [74]. *ACE2* transcript levels were remarkably increased in stomach adenocarcinoma and colon adenocarcinoma patients, which was also corroborated with the observations stated earlier. Besides this, *TMPRSS2* was overexpressed in colon adenocarcinoma and prostate adenocarcinoma but decreased in breast cancer, a similar observation to those made by Li *et al.* and Katopodis *et al.* [75,76].

Based on Kyoto Encyclopedia of Genes and Genomes analysis, *ACE2* and its 100 co-overexpressed genes show their effect on fat digestion and absorption, phenylalanine metabolism, renin–angiotensin–aldosterone system, tryptophan metabolism, vitamin metabolism and steroid hormone biosynthesis [76]. Carcinogenesis and viral infections both are proficient to regulate NAMPT/NAD signaling. Studies indicate links between the NAMPT/NAD and renin–angiotensin–aldosterone system metabolic cascades which may support the crosstalk between cardiac functions, lung dysfunction and this infection [77–79]. These observations indicate that those suffering from cancer are more susceptible to this infection [80].

Changes in the levels of TMPRSS2, ACE2 and CTSL/B have been studied in multiple cancer samples from The Cancer Genome Atlas. However, the ways in which they are regulated in multiple cancer types have not been studied yet. Currently, researchers and clinicians need to come forward and work in a collaborative manner to determine how their thresholds are changed at the molecular level in multiple cancer scenarios, and to establish how we can target them to treat cancer patients affected with COVID-19.

## Comorbidity of diseases: an epigenetic scenario

‘Epigenetics’ refers to the genetic expressivity and expression of phenotypic traits in response to environmental cues. The epigenetic scenario includes the multitude of ways to modify DNA conformation and access or denial to the transcription factors through epigenetic modifications like DNA methylation, histone methylation, acetylation, deacetylation and so on. The outbreak of the SARS-CoV-2 pandemic and its clinical presentation have been linked with epigenetic modifications responsible for viral entry regulation, mechanisms of immune evasion and cytokine-induced inflammatory responses [81]. The epigenetic scenario plays a modulatory role in COVID-19 severity and the occurrence of comorbidities in terms of respiratory diseases, cardiovascular complications and diseases like diabetes or cancer. These diseases result from complications arising from the COVID-19 infection. Furthermore, they show a reciprocal relationship with COVID-19 in terms of elevated vulnerability and chances of infection due to an intrinsically immunocompromised state or aberrant cellular processes resulting from COVID-19-linked epigenetic changes [82,83].

SIRT1, a histone deacetylase (HDAC) and a notable epigenetic regulator, has been studied for its function in the modulation of the epigenetic landscape manifested under COVID-19. It is effectively studied in the lungs of patients with severe COVID-19 comorbidities [84]. Upon stress, SIRT1 can epigenetically regulate the ACE2 receptor, instrumental in binding with the S protein of this virus. Furthermore, treatment with NSAIDs inhibits the activity of SIRT1 and affects *ACE2* expression. Different studies report a dual role of SIRT1 as both a tumor suppressor and tumor promoter in cancer depending upon the target signaling pathways and localization. Aberrant SIRT1 levels have been associated with cancers including acute myeloid leukaemia, melanoma and primary colon, prostate and breast cancers. Other comorbidities such as metabolic diseases, diabetes, cardiovascular disease and chronic respiratory syndromes are all associated with decreased SIRT1 levels. Therefore an epigenetic modulator like SIRT1 acts as a coordinating link between COVID-19 and its allied comorbidities [85,86]. Chromatin-modifying epigenetic mechanisms also play a role in the remodeling of pulmonary tissues, which in turn leads to respiratory complications like asthma and chronic obstructive pulmonary disease. Such comorbidities increase the risk of COVID-19 infection [84]. Histone acetyltransferases and BRD4 acetylate histones, while HDACs remove acetyl groups from histones. Differentially methylated regions exist around the promoters of interferon-related genes. They are hypomethylated in patients with COVID-19 and can often help in understanding the epigenetic roles of the methylation process in SARS-CoV-2 infection compared with uninfected scenarios. For instance, qRT-PCR revealed the higher expression of *IFI27* and *OAS2* in patients with COVID-19 compared with unaffected patients, implicating their crucial role in accelerating infection [87–89]. Additionally, epigenetic regulation via noncoding RNAs regulates the inflammation. All of these epigenetic landscape-based modifications end with the production of proinflammatory cytokines and chemokines, elevation of the neutrophil-to-lymphocyte ratio and coagulation; finally, it results in lung diseases, COVID-19 vulnerability and, in the most severe scenario, lung cancer. Considering the crucial connections between inflammatory comorbidities, lung cancer and COVID-19, it has been suggested that immune responses may be identified by the ectopic expression of miRNAs. Several miRNAs have been found to target COVID-19-related ACE2 or TMPRSS2 host cell receptors. For instance, lung cancer progression and metastasis are suppressed by miR-1207 through targeting CSF1, which in turn gets targeted by the competitive binding of SARS-CoV-2 RNA. The miR-200 family and their tumor suppressive action in multiple cancers along with their regulatory role and overexpression of *ACE2* highlight their strong candidature as an epigenetic modifier linking COVID-19 and cancer [89–91].

A comprehensive study about the contributions of epigenetic mechanisms to COVID-19 and allied comorbidities including cancer can help in identifying emerging epigenetic biomarkers for COVID-19 and pave the way for promising therapeutic targets to combat the progression of the disease states.

## Host genetic & epigenetic machineries in COVID-19 & their induction in cancer patients

Several host epigenetic changes have been noted in SARS-CoV-2-infected cancer patients. To understand this, knowledge about the host genetic and epigenetic landscape is crucial. In this section we discuss the COVID-19 host genetic and epigenetic landscape, and epigenetic changes induced in cancer patients with this infection.

### Host genetics

In addition to environmental and social factors, the individual genetic make-up of the host also determines the vulnerability to, severity of and immunological response to this viral infection. Identification of host genetic factors might help in the study of therapy-oriented biological mechanisms [92,93], and the genetic make-up of the host can be studied to measure the severity and impact of the disease on human health [94]. Recently, the detection of single-nucleotide variants and short indels has been performed by whole-exome sequencing or short-read sequencing data generation [95], but these findings were not able to identify the multiple SARS-CoV-2-related variants. However, to resolve this issue, several advanced technologies have been utilized, such as optical genome mapping using BioNano’s Saphyr^®^ system (BioNano Genomics, CA, USA). By this technology, SARS-CoV-2-associated structural variants were identified in 37 hospitalized infected individuals [96]. Furthermore, the variants’ specific small or rare structural changes were analyzed in a similar group of patients by whole-genome sequencing analysis. The identified structural variants were compared with the normal population by calculating the ratios to top-ranked genes. This study determined and confirmed the direct impact of some genes on the severity of disease in infected patients, and concluded that optical genome mapping is an efficient method to determine SARS-CoV-2 structural variants that help in the determination of disease outcome and mortality [96,97]. Recently, a patient-center-based investigation has identified 38 genes which are directly linked to different host–viral association pathways: innate/inflammatory immune responses, passage resistance, viral propagation, RNA editing and viral spread. This study identified seven types of SARS-CoV-2 structural variants, affecting 31 genes, in nine out of 37 patients by using the optical genome mapping platform. Of the affected genes, *STK26* and *DPP4* are on the top of the list and highly promising genes. Furthermore, the application of a population frequency filter of less than 20% was performed in the Bionano control dataset, which revealed that seven genes in 21/37 patients were highly involved, with four structural variants. All these findings suggest the clinical importance of these identified genes and their effects on infection, immunological response and replication pathways [98–100].

Genome-wide assay-based identification of the chromosome locus 3p21.31 has also been studied in patients with severe SARS-CoV-2 infection and respiratory failure. This locus includes a gene cluster that contains several genes such as *LZTFL1*, *SIT1*, *FYVE* and *FYCO1*, *CCR9*, *CXCR6* and *XCR1* [101–103]. This locus was determined in Host Genetics Initiative (HGI) meta-analysis to be significantly involved in disease severity and infection susceptibility. The COVID-19 HGI maps the human genetic structure as it relates to SARS-CoV-2 [101,104,105].

Late detection of SARS-CoV-2 RNA and recurrence of this infection is mostly reported in recovered patients [106]. Researchers have investigated the chances of genomic association of SARS-CoV-2 in human cells, which may be responsible for false-positive PCR results [107]. The transcription of large viral component sequences was observed in some infected patient tissues where integration of host genomic sequence has happened; thus this possibility of genomic integration contributes to the false-positive results upon reinfection [106,108–110].

Genetic vulnerability to SARS-CoV-2 infection was further studied by probing DNA polymorphisms in *ACE2* and *TMPRSS2* from approximately 81,000 human genome samples. Unique genetic vulnerability patterns were observed in both *ACE2* and *TMPRSS2* across several types of patients. However, the *ACE2* polymorphism is specifically studied to be involved in the cardiac as well as the pulmonary system by inhibiting the angiotensinogen–ACE2 interaction. An *ACE2* polymorphism (p.Arg514Gly) was observed in African and African–American populations which inhibits the angiotensinogen–ACE2 interaction. Moreover, a unique type of polymorphism (p.Val160Met) was also observed in *TMPRSS2*, demonstrating that there are different type of genetic vulnerability for this infection and also for the tumor presence and major risk to male infected individuals. These polymorphisms in *ACE2* or *TMPRSS2* may also be used to direct SARS-CoV-2 therapies [111–114].

The deeper understanding of host genetics is a crucial factor for disease susceptibility and severity. It helps in understanding of the viral pathophysiology, infection and in the discovery of novel drug targets against this infection. It also helps in the prediction of several elements such as disease risk factors, outcome and in diagnostic biomarker identification. Moreover, the concept of host genomic integration of SARS-CoV-2 is not resolved yet. Hence deeper and more focused clinical investigations are required to make progress in this field.

### Host epigenetic machineries

**Figure 4.**
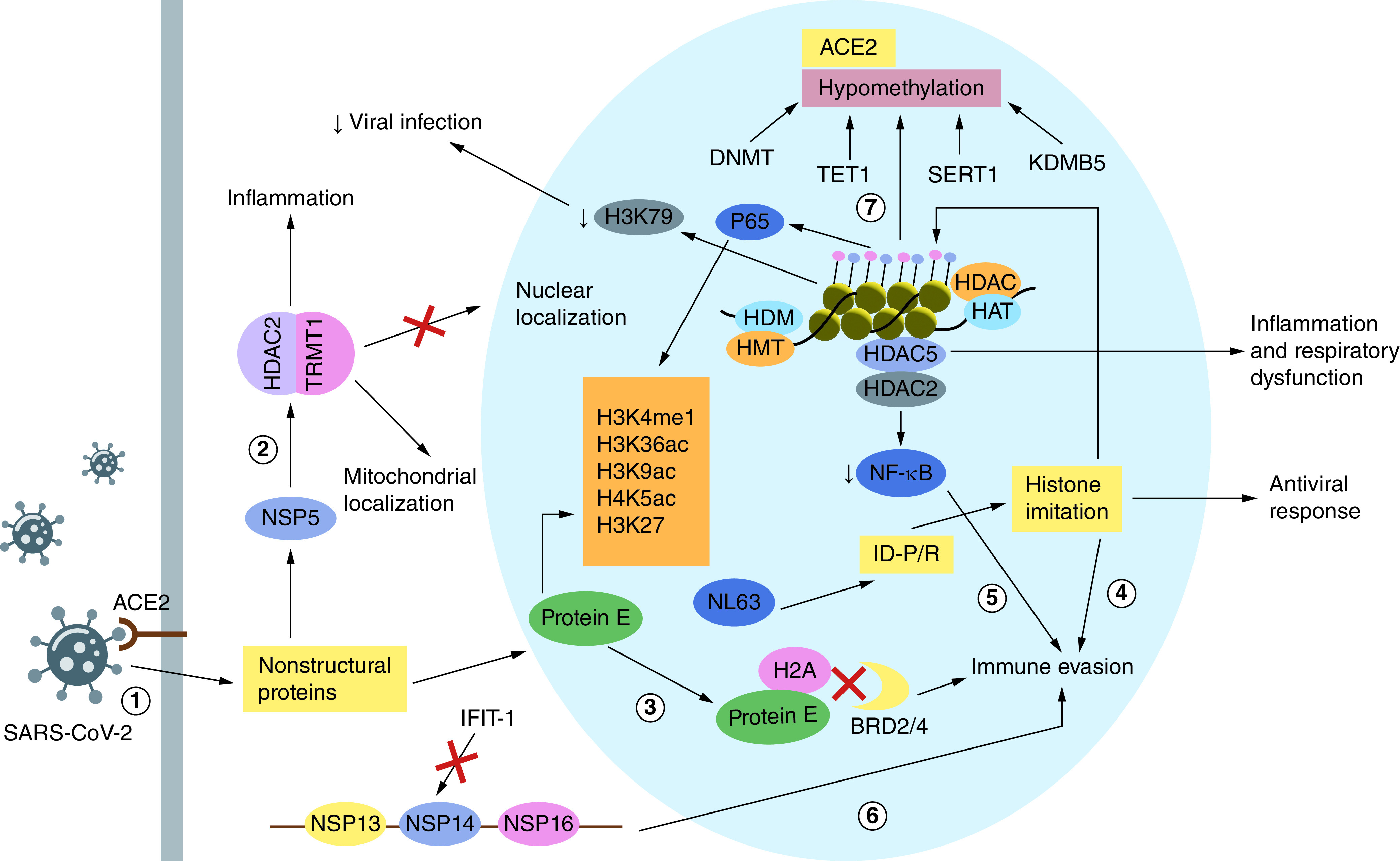
Associations and alterations of SARS-CoV-2 in the host epigenetic machinery.

Viral entry in this infection mostly occurs via the ACE2 receptor. Host epigenetic changes (hypomethylation) at CpG sites across *ACE2* in pulmonary epithelial cells provide the first step for viral entry [44,115]. However, methylation of the *ACE2* promoter is controlled or regulated by HDAC2, DNMTs (DNMT3a and DNMT3b), TET1, KDM5B and MAX [44]. After interaction with the ACE2 receptor, viral particles make their entry into the host cell via the endocytosis pathway [116] and are able to release their RNA in the cytosol, which acts as a template for negative-strand synthesis. Subsequently, this causes the production of viral mRNAs and several viral proteins. Synthesized NSP5 proteins were found to interact with both HDAC2 and TRMT1 that inhibit their entry to the nucleus [44,115]. Furthermore, SARS-CoV-2 protein component E has a lot of similarity with histone 2A (H2A). Due to this similarity, protein E is able to prevent the interaction of both H2A and BRD2, which leads to immune evasion (Figure 4) [117,118].

**Figure 5.**
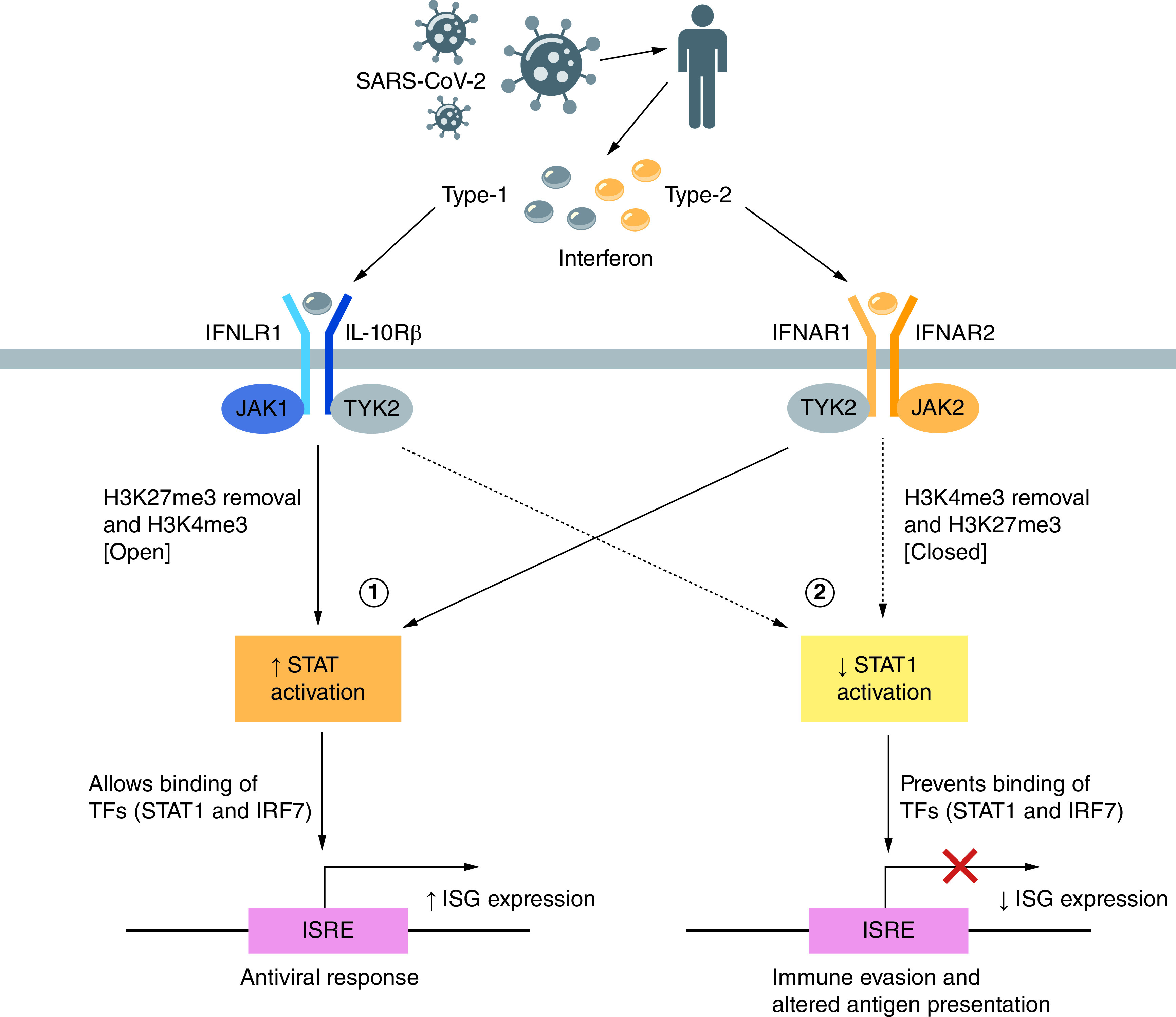
Type I and II interferon-mediated inflammatory immune responses. The figure represents both chromatin open and closed states, which result in antiviral immunity and immune evasion along with altered antigen presentation, respectively.

This infection in host cells induces type I and III interferons via eliminating the methyl group from H3K27me3-repressed histones and activating methylation (H3K4me3) at the histones. Through this elimination and activation process, the chromatin is shifted to an open state from its closed conformation, which further leads to the induction of ISG expression after the incorporation of multiple transcriptional factors including STAT1 and IRF7. On the other hand, incorporation of repressive histones (H3K27me3) can condense the chromatin (closed state) that prevents the binding of transcription factors, leading to the reduction in ISG expression that results in the induction of immune evasion (Figure 5) [84,117,119].

Human coronavirus NL63 derives the intrinsically disordered proteins or regions which have been studied and found to interact with short linear motifs (SLiM) proteins. A few residues of host SLiM proteins are quite similar to those of histone proteins and are also capable of histone mimicry, which will ultimately contribute to antiviral immunity and immune evasion [44,120]. Furthermore, nuclear histone HDAC5 is reported to induce anti-inflammatory immune responses through the stimulation of proinflammatory cytokines [44,84]. However, HDAC2 activation increases in this infection, which causes altered NF-κB activity and results in the inhibition of monocyte and macrophage functionality [117,121]. ORF1 of viral RNA contains NSP13, 14 and 16; NSP13 is responsible for its 5′-triphosphatase and helicase activity, NSP14 possesses guanine N7-methyltransferase activity, and NSP16 is responsible for 2′-ortho-methyl transferase activity [44,115]. Furthermore, IFIT-1 associates with the viral RNA at its 5′ end, where the viral RNA lacks 2′-ortho-methylation, and results in inhibition of the translation process of viral RNA [122–124].

Generally, removal of H3K27me3 and activation of H3K4me3 at histones opens the chromatin and leads to increases in ISG expression and antiviral immune responses. According to the present knowledge and our understanding, targeting of this infection at the epigenetic level is also possible. Synthesis and searching of existing libraries of small molecules which hold the chromatin in an open state that leads to the development of an antiviral immune response may mean that, in the near future, researchers and clinical investigators can focus and work in this field to target this deadly infection at the epigenetic level.

### Induced epigenetic changes in cancer patients with COVID-19

Modulation of host epigenetics by this deadly virus – the systematic manipulation and exploitation of the host nuclear structure for its own propagation – has emerged as an interesting area of research. Epigenetics is largely the combinatorial study of both genetic and nongenetic factors that control phenotypic fate and are affected by external and environmental stimuli. These stimuli manipulate genetic activity without altering the DNA code. In virus–host interactions, existing data indicate that viral agents can alter host epigenomes through methylation/acetylation of DNA, mRNAs, chromatin remodeling, noncoding RNAs and histone changes to maintain the gene expression profile of the host and then recruit the host transcription system to favor viral propagation and transcription. Coronaviruses have extensively evolved to use these processes. The virus’s interference with the host epigenetic machinery regulates the immune response, aiding its replication and pathogenesis. The epigenetic modifications enable the viral particles to enervate the host immunological pathways to proliferate the infection. Most of these signaling pathways have been investigated as newer therapeutic targets. Histone modifications and DNA methylation/acetylation lead to chromatin remodeling and, in combination with the other modifying noncoding RNAs (miRNA, siRNA, lncRNA) and proteins (sirtuins, prions etc.), make the chromatin accessible to regulatory proteins in the transcription process. Enzymes that manifest epigenetic regulations are HDACs, histone acetyltransferases, kinases and histone methyltransferases that work directly on the structural components of chromatin. On the other hand, enzymes like thymine DNA glycosylase, ten-eleven translocation proteins and DNMTs are involved in dynamic methylation/demethylation of DNA, thereby regulating gene expression [125,126].

#### ACE2

**Figure 6.**
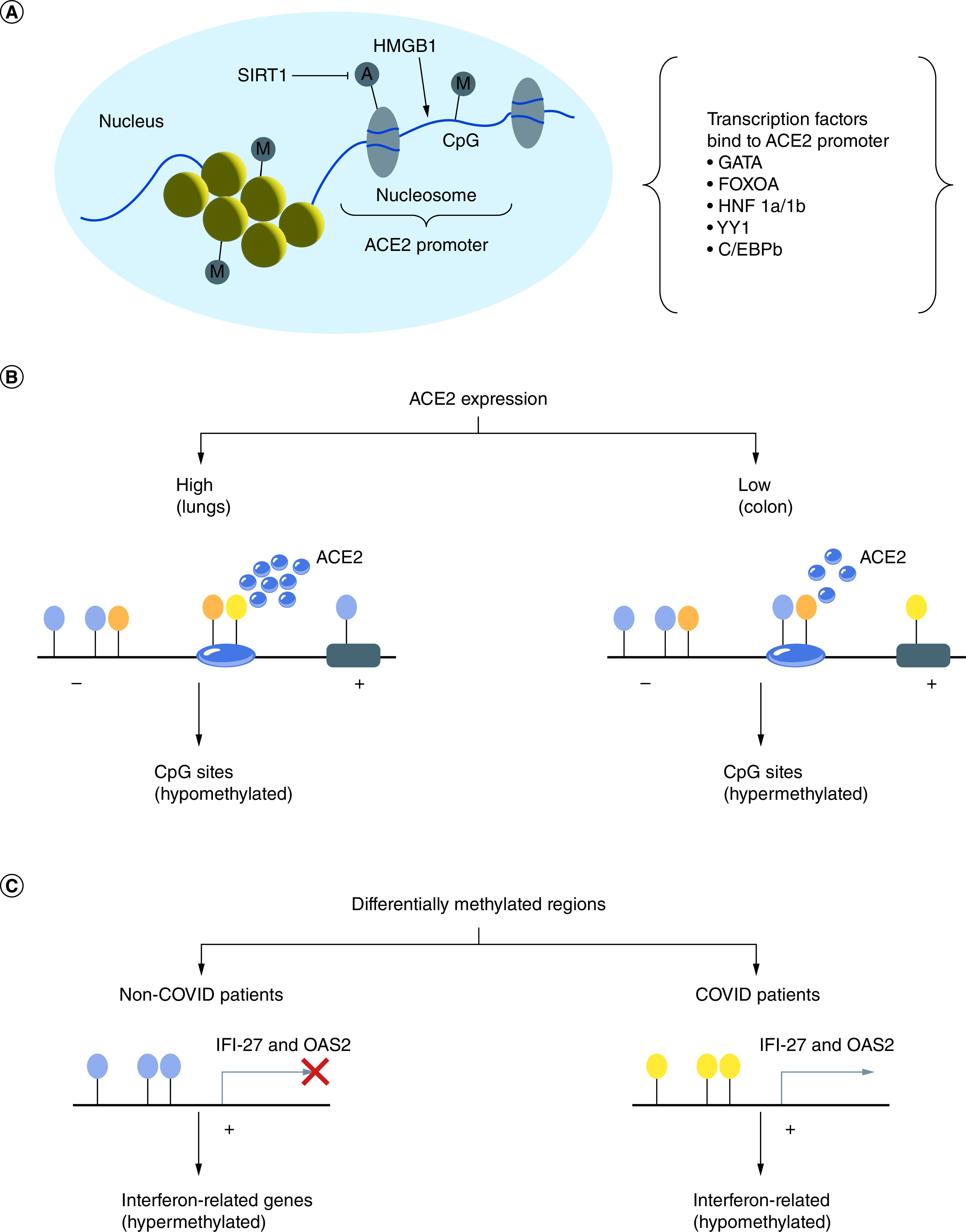
Effect of DNA methylation and chromatin establishment on *ACE2* expression in SARS-CoV-2 patients. **(A)** Transcription factors able to associate with the *ACE2* promoter. **(B)** Methylation status of the *ACE2* promoter in both higher and lower *ACE2-*expressing tissues. **(C)** Comparative methylation status of infected and non-infected individuals.

Genome-wide DNA methylation array and chip methylation pipeline studies reflect a diverse range of DNA methylation in *ACE2* among various tissues. In one such study, a much lower amount of *ACE2* methylation was observed in three CpG locations (cg04013915, cg08559914 and cg03536816) in pulmonary epithelial cells than in control tissues [127]. A successive study reported that *ACE2* hypomethylation is mostly restricted to females in comparison to males, indicating involvement in AT-II metabolism and participation with genetic or hormonal level changes [128]. However, transcriptomics-based analysis showed a lack of relationship of *ACE2* with patient-specific factors such as gender, race or age. Furthermore, smokers of Asian origin exhibit an elevated expression of *ACE2* compared with nonsmokers, suggesting an epigenetic consequence in the pulmonary system [129,130]. However, the confirmation of this perspective necessitates the acquisition of proteomic-based data. In another study using four distinct public databases for lung tissues, *ACE2* hypomethylation indicated the lungs have greater responsiveness for this infection (Figure 6) [131].

Exploration of oncology-based databases disclosed that the promoter of *ACE2* is hypomethylated and that its mRNA is greatly expressed in stomach, colon, pancreas, rectum, kidney and lung cancers [132]. Further study suggested a role of TNF-α in modulating the transcription of *ACE2* and an association with disease complications in endothelium cells. This information suggests that TNF-α may elevate DNA methylation of the promoter region of *ACE2* by reducing the potential of DNMTs (TET1, DNMT3a and DNMT3b) [133]. Systems biology and transcriptomics-based approaches divulged the notable correlation of high *ACE2* expression with *KDM5B*, *HAT1*, *HDAC2* and *RAB1A* in patients with other comorbidities (chronic obstructive lung disease, diabetes and hypertension) [134]. Also, the SIRT1- and NAD+-dependent HDACs play important roles in the activation of the *ACE2* promoter, suggesting that SIRT1 may act as a possible epigenetic drug target for therapy against SARS-CoV-2 infection [135]. Pathway enrichment analysis also suggests the crucial responsibility of KDM5B to regulate multiple other genes that are linked with *ACE2*, feasibly via acetylating and methylating epigenetic biomarkers (H3K27ac, H3K4me3 and H3K4me1). The present literature about COVID-19 association with ACE2 receptor epigenetic pattern is very less. However, the underlying molecular mechanism of *ACE2* promoter activity cannot be diminished during this ongoing pandemic as the pathogenesis is established [35,135,136].

Recently, CpG-targeted probes were used for the promoters of *ACE2* and *CTSL* in stomach adenocarcinoma and pancreatic adenocarcinoma, respectively, to investigate the effect of epigenetic alterations at the transcriptional level [137]. A combinatorial study with Methylation450k profiles and the DiseaseMeth database showed that in pancreatic adenocarcinoma the DNA methylation of *CTSL* was notably less in comparison with stomach adenocarcinoma; both were normalized against physiological tissues [138]. Moreover, *ACE2* methylation in stomach adenocarcinoma and pancreatic adenocarcinoma were also studied for their negative correlation using the DNA Methylation Interactive Visualization Database analysis [139]. Analysis of the UALCAN database using the GEPIA tool showed the correlation of *ACE2* expression with its methylation profile. Mostly, a solid and well-established inter-relationship between the expression of genes and their promoter methylation status, in accordance with the discoveries relating to *ACE2*, was observed in multiple cancers. *CTSL* has a greater β-value in rectal adenocarcinoma and colon adenocarcinoma, which results in its lower expressions; however, the promoter methylation status is lower in stomach adenocarcinoma, which can also lead to its greater expression [140].

#### T cells

Recently, a study was performed to understand the functional impact of a genetic vulnerability locus related to SARS-CoV-2 at chromosome 3p21.31 in immune cells (HL-60, THP-1, K562 and Jurkat cell lines, which resemble human promyelocyte, monocytes, erythroid cells and T lymphocytes, respectively) [141]. *SLC6A20* and *CCR9* were reported to be probable target genes in the SARS-CoV-2 locus using CRISPR/Cas9 oriented genomic excision. Six fine-mapped variants (1834326463, 1876374459, 1573064425, 1813081482, 1835652899 and 1535044562) overlapping with human T-cell particular primed enhancers were found. This discovery indicates that the mechanism of epigenetic regulation may hamper the function of T cells in this infection [141]. A further study noted that even after 5 months of infection, cross-reactive CD4^+^ T cells are still found in 70% of the convalescent infected population [35].

#### IL-6

IL-6 can modulate the functions and expressions of several genes by blocking DNMTs (DNMT1 and DNMT3B) [43]. The *IL-6* gene is observed to be activated in DCs through acetylation of the KLF4 transcription factor in inflammation-induced ARDS [142,143]. An amalgamation of epigenetic acetylation and phosphorylation in herpes virus encrypts IL-6 and sufficiently promote STAT3 activity [144]. All these earlier reports and well-established literature build up the hypothesis that the expression of IL-6 can be epigenetically hampered in SARS-CoV-2 infection. Assay for transposase-accessible chromatin (ATAC) and Chip-Seq datasets from 157 and 839 cell/tissue varieties of mouse and human origin were utilized to test and validate such assumption via ENCODE [145]. The research group established the remarkable relative presence of both H3K27ac and H3K4me3 marked in the promoters of *ACE2* and *IL-6* genes of several human and mouse specimens. These types of epigenetic changes were studied extensively in human in comparison to *Mus musculus* orthologs. In the distal and proximal areas of the human *ACE2* promoter, the presence of two- to three-fold more histone changes indicated its much greater activation and transcription compared with its *Mus* *musculus* ortholog. On the basis of these discoveries, upregulated expression of non-ISGs (*IL-6* and *ACE2*) may act as a specific signature for the inflammation originating from this infection, which can unbalance the physiology of this gene in the renin–angiotensin–aldosterone system [146].

#### Interferon-stimulated genes

Several pieces of evidence suggest that the production of functional cytokines and chemokines which are anti-inflammatory in nature controls the virus replication and propagation by promoting antigen presentation [147–149]. The expression of ISGs takes part in the prevention of any viral infection by promoting effective immunological function. A series of signaling cascades is stimulated by the type-1 interferons which leads to transcription of multiple anti-inflammatory ISGs [150,151]. Knowledge of this process is limited in relation to SARS-CoV-2 infection; however, results from respiratory RNA viruses and influenza suggest that SARS-CoV-2 and MERS-CoV pathogens significantly slow down ISG expression in the host. Proteomics and transcriptomics-dependent studies in Calu3 cells reported the varied expressions of specific ISGs. Calu3 cell-based development of this infection suggests the higher stimulation of ISGs effectors; however, this reaction was remarkably slowed in respect to higher expression after 48 h of infection. A study in 2012 showed that MERS-CoV infection produced a dramatically lower release of ISGs after 18 h of infection, in response to the reduced expression of probable ISG subsets [135,150]. Further studies support the concept that inhibition of ISGs is not due to any problem in the signaling cascade but occurs due to the histone modifications caused by the infection. The host system induces type I and III interferon upon viral infection, which leads to the activation of the chromatin modification complex and results in the removal of the H3K27me3 repressive histone and activation of H3K4me3. This change from the inactive to the active state of histones permits the association of some transcriptional components (IRF7 and STAT1) which further leads to the induction of ISG expression [152,153]. However, the inclusion of H3K27me3 repressive histone modification and exclusion of functional H3K4me3 may condense the chromatin (closed state) and hinder the binding of these transcriptional factors, leading to inhibition of ISG expression. Additionally, the enzyme Dot1L is connected with diminished antiviral immune responses and promotes viral replication and propagation through inhibition or downregulation of H3K79 methylation, a crucial antiviral activity [154,155]. The expression of ISGs and the level of H3K9me2 within the type I interferon promoter are correlated inversely in DCs, indicating that histone changes are an essential controller of the interferon response [8]. The divergent tendency of histone marks in the ISG promoters were investigated in one study by chromatin immunoprecipitation (ChIP)–PCR; the findings suggested that the ISG promoter areas contain a number of histones along with the functional sites of H3K4 monomethylation rather than suppressive H3K27 trimethylation, resulting in an open chromatin state and thereby promoting active transcription and expressions of ISGs during the infection [156]. In contrast, MERS-CoV infection showed augmented H3K27me3 levels and lower H3K4 trimethylation on the promoters of specific ISGs, which were not upregulated [157].

#### Histone deacetylases

Respiratory illnesses are directly linked with SARS-CoV-2 infection and are aggravated by the significant inflammatory immune responses induced by macrophages and monocytes. Monocytes act in innate immunological responses and generally migrates to the infected areas, differentiate into macrophages and further proceed to the generation of several defensive proinflammatory cytokines and chemokines which help in cell migration [158]. Histone modifications (acetylation and de-acetylation) play a remarkable role in the activation and survival of macrophages. HDAC-mediated regulation of non-histone proteins is also pivotal for several physiological roles (replication and DNA repair as well as HIF-1α, p65, STAT3 and p53 signaling). Macrophagic HDAC exerts its proinflammatory roles by inducing several proinflammatory cytokines (IFN-γ, IL-1α, MCP-1, IL-1β and TNF-α) that can be controlled by HDAC inhibitors [159]. An HDAC5 overexpression study indicated that stimulation of MCP-1 and TNF-α in macrophages further produces these proinflammatory immunological responses. Moreover, the function of HDAC2 in regulating NF-κB suggests its prominent role in immune evasion. The occurrence of HDAC2 in the nucleus makes it more capable of preventing NF-κB, which results in functional manipulation of macrophages and monocytes [160]. Identification of viral proteins and their associating host partners could suggest newer insights into the molecular basis of such immune modulation. Recently, protein 3b of SARS-CoV-2 was reported to associate with RUNX1b, resulting in the stimulation of transcription of several genes involved in T-cell differentiation and hematopoiesis [158]. In the immune response to this infection, protein 3b is known to encourage the ERK pathway-based phosphorylation of RUNX1b and further triggers the cytokine IL-2 [161]. This high-grade molecular association of RUNX1 is known to be mediated by HDAC appointment and killer T-cell response [162]. These studies strongly support the operation principle of effective T cell for amplification of immunological responses can be epigenetically regulated.

#### Bromodomain-containing proteins for evading the host immune response

There is a conserved system of histone-related proteins, and the histone acetyltransferases play a vigorous role to control the transcription of chromatin-associated genes. Bromodomains (BRDs) particularly associate with the acetylated histones and modulate the expression of several genes [163]. Recently, an affinity chromatography-linked mass spectrometry study revealed the association of protein E with the BRD subclass of proteins (BRD2 and 4), leading to the prevention of BRD histone functions by imitating the histone structure. Histone 2A shows sequence similarity with the transmembrane fragment of protein E, which possesses inhibitory activity over histones which removes their association with BRD2 and leads to the invasion of host immunological responses [164].

Assessment of both wild-type and catalytically dead mutant (C145A) of Nsp5 revealed that wild-type Nsp5 shows a higher correlation confidence with HDAC2 that anticipates a breakage point between nucleus localized sequence while HDAC domain reveals opposite impact. Interactive exploration suggested the harmony of mutant Nsp5 with TRMT1 and wild type Nsp5 with both TRMT1 and HDAC2. Besides this, Nsp5 detaches the zinc fingers for nuclear localization of TRMT1 and regulates its mitochondrial localization [165]. A similar study using chemoinformatic data indicated that apicidin and valproic acid cause HDAC2 inhibition at 120 and 5 nM, respectively; other compounds like ABBV-744 and CPI-0610 had affinities of 2 and 39 nM on BRD2 and BRD4, respectively [44,166].

Limited reports and research in the field of induced epigenetic changes in SARS-CoV-2-infected cancer patients suggest a need for more focused clinical investigations to reproduce and confirm these changes. A few epigenetic changes have been listed above; targeting them with the development of specific inhibitors may help to control this infection and its impact on cancer patients. In the near future, basic research and development of inhibitors in this area may provide hope for the treatment of SARS-CoV-2-infected cancer patients. In conclusion, researchers, scientists, clinicians and pharmaceutical industry workers have to come forward and work in a collaborate manner to achieve this objective for the welfare of society and humanity.

## Conclusion & future perspective

As the world is yet to recover from the damage caused by the COVID-19 pandemic, the recent emergence of more infectious, mutant viral strains continues to adversely influence human lives globally. Moreover, in the face of COVID-19, it has become extremely difficult to address the needs of cancer patients due to the complexities involved in these two critical diseases. Cancer patients show immunocompromised traits and greater susceptibility to viral infections. This has triggered a plethora of research to be directed toward delineating the correlation between the two diseases as well as to finding suitable therapeutic strategies to address the scenario. This infection is keenly linked with genetic and epigenetic modifications that influence the expression profile of ACE2 and other immunoregulatory genes. However, the differentially methylated CpG sites of various promoters encoding immunoregulatory genes act as SARS-CoV-2 epigenetic signatures having no response to enhanced viral infections. Differential epigenetic regulation involves DNMTs, KDM5B and SIRT1 to modulate the epigenetic mark H3K27me, resulting in the subsequent cytokine storm which leads to the complex pathophysiology of the disease.

The histone modifications and similar trends shown by molecular players associated with both diseases provide insights into their association. Moreover, detailed understanding of the linkage between the two diseases is needed as further mechanistic evaluation of the epigenetic scenario can help to shed light on the occurrence of comorbidities of diseases such as cancer and COVID-19. Discovery of epigenetic biomarkers associated with these comorbidities is important to design suitable future therapeutic interventions.

**Executive summary**

- SARS-CoV-2 has an immense impact on cancer patients’ prognosis, as inflammatory immune responses induced by this infection promote cancer aggressiveness.
- ACE2, CTSL/B and TMPRSS2 receptors play dual roles in COVID-19 and many cancers (lung, head and neck, colon, glioma, prostate, breast, renal, pancreatic and other carcinomas).
- SARS-CoV-2 infections have been found to be more intense in cancer patients than in normal individuals.
- Epigenetic scenarios can help to shed light on the occurrence of comorbidities of SARS-CoV-2-infected cancer patients.
- Modulation of the host epigenetic landscape is a key hallmark in both cancer and viral infections, including COVID-19.
- Basic and translational research on this comorbidity status may provide hope and aspiration for better treatment regimens.
- Scientists, clinicians and pharmaceutical companies have to come forward and work in a collaborative manner to achieve this goal for the welfare of society and humanity.
